# Barrier or breach? Assessing swine housing features for mosquito threats

**DOI:** 10.3389/finsc.2025.1606259

**Published:** 2025-09-04

**Authors:** Christy J. Hanthorn, Lee W. Cohnstaedt, Natalia Cernicchiaro

**Affiliations:** ^1^ Center for Outcomes Research and Epidemiology, College of Veterinary Medicine, Kansas State University, Manhattan, KS, United States; ^2^ United States Department of Agriculture, Agricultural Research Service, National Bio- and Agro-Defense Facility, Foreign Arthropod-borne Animal Disease Research Unit, Manhattan, KS, United States

**Keywords:** mosquito, biosecurity, swine, swine housing, integrated mosquito management plan

## Abstract

Effective mosquito control is critical in swine production to reduce disease transmission and prevent mechanical damage. However, current biosecurity measures on swine premises primarily target microbial pathogens, often overlooking the importance of excluding insects, particularly mosquitoes. While vector-borne disease transmission is the primary concern, mosquito infestations also contribute to mechanical damage, leading to secondary infections, stress-related production losses, and compromised animal welfare. Mitigation efforts aimed at mosquitoes can also have broader benefits by reducing other insect pests that compromise swine health. Despite the availability of tools and strategies for mosquito monitoring and control, standardized protocols and evaluations of effectiveness remain limited. This study aims to assess the protective attributes of swine housing against mosquito threats and identify vulnerabilities that may increase the risk of insect-borne diseases. By understanding these factors, targeted biosecurity strategies can be developed to enhance insect exclusion and reduce the overall impact of mosquito infestations on swine health and production. A key focus of this assessment is the reduction of mosquito populations within and around swine housing facilities. By providing swine producers and veterinarians with actionable insights and practical mitigation strategies, this study seeks to strengthen mosquito management efforts, ultimately improving herd health, productivity, and overall biosecurity.

## 1 Introduction

Historically, swine in the United States (U.S.) were kept in extensively managed outdoor housing systems where they faced conditions detrimental to production including parasite burdens, predation, and suboptimal environments (1–3). While many pigs on small^1^ swine farms are still housed in a manner that allows them access to the outdoors^2^ (4), the majority of large^1^ swine farms house their pigs in total confinement with mechanical ventilation^3^ to accommodate more intensive management strategies such as the use of artificial insemination and early weaning (2, 5). The U.S. commercial swine industry’s transition away from outdoor housing to indoor confinement facilities relieved many adverse production pressures exemplified by an overall decrease in parasite burden to the point where many pigs raised indoors do not receive routine treatment for parasites. This creates a potential vulnerability against ectoparasites and the diseases they spread. Environmental conditions and weather events intermittently create suitable conditions for insect pests to cause problems on all types of swine farms, including sites with intensively managed swine (6, 7). Due to the variability in facility design, even swine facilities classified as “total confinement” do not preclude the entrance of insects.

Insect bites can result in mechanical damage from a pig’s primary inflammatory response or secondary excoriation from scratching (8, 9). Additionally, insects can act as mechanical or biological vectors for swine pathogens (**Supplementary Table S1**). Examples include swinepox, transmitted mechanically by the pig louse, flies, and mosquitoes, and vesicular stomatitis virus (VSV), transmitted either biologically or mechanically by hematophagous flies specifically biting midges (*Ceratopogonidae*), sand flies (*Phlebotominae*), and black flies (*Simulidae*). Mosquitoes are also biological vectors of zoonotic Flaviviruses including Japanese encephalitis virus (JEV) and West Nile virus (WNV), and Bunyaviruses such as Rift valley fever virus (RVFV) which can infect both pigs and humans (9, 10).

Biosecurity is defined in the World Organization for Animal Health’s Terrestrial Animal Health Code as, “a set of management and physical measures designed to reduce the risk of introduction, establishment and spread of animal diseases, infections or infestations to, from, and within an animal population” (11), and is critical for preventing disease spread among both individual swine and swine premises (i.e., the entire area of a site including swine housing facility and the surrounding land) (12–14). Biosecurity guidance to prevent bacterial and viral pathogen introduction onto swine premises (i.e., bioexclusion) is well-developed (12, 15–17). However, recommendations for protecting pigs from insects remain generalized and rarely address specific problematic species. Recommendations of effective biosecurity measures against mosquitoes in swine populations under different housing conditions are still lacking (15, 16, 18–20). While biosecurity resources exist for swine producers, they often underemphasize insect exclusion compared to microbial exclusion (15, 16, 19, 21). Conversely, insect management resources are available but are not explicitly framed as biosecurity measures (20, 22). Although mosquito-vectored diseases are currently rare in the U.S. swine industry, effective mosquito biosecurity would be critical in preventing and managing outbreaks in the event of a foreign animal disease incursion, as demonstrated by the challenges Australia’s swine industry faced following the recent emergence of JEV (23, 24).

In this perspective manuscript, we highlight the seasonal burden posed by insects, specifically mosquitoes, on swine farms and examine how structural and management features of swine housing influence mosquito presence and access. We place special emphasis on identifying design-related vulnerabilities and proposing practical, evidence-based recommendations to reduce mosquito contact with humans or animals. To address insect-related biosecurity gaps, we explore incorporating Integrated Mosquito Management (IMM) strategies into existing enhanced biosecurity site plans (25). Incorporating IMM allows swine producers to evaluate and adopt surveillance and management tools best suited to their facility design, operational capacity, and environmental conditions. Because complete mosquito bioexclusion is likely unachievable, producers must weigh intervention costs against potential benefits using targeted mosquito surveillance data (to assess needs) and swine production records (to measure impacts). Our primary aim is to assess swine housing attributes that either minimize mosquito contact with pigs, or inadvertently facilitate mosquito entry, considering vector physiology, behavior, and habitat requirements. We also review current guidance on limiting mosquito access to swine facilities, reducing pig-vector interactions, and preventing the establishment of resident mosquito populations. While the emphasis is on mosquitoes, we include relevant comparisons with other flying insect pests that share similar pathways of entry and impact on swine health, to broaden the applicability of the suggested strategies.

## 2 Mosquito physiology and behavior

Both male and female adult mosquitoes require sugar meals (e.g., nectar, rotting fruit, livestock feed, or animal feces) for energy to fly and mate. Additionally, females typically need a blood meal to produce and lay eggs (26). To locate these resources and thrive in diverse habitats, mosquitoes have evolved specialized behaviors and sensory adaptations. Adult mosquitoes are highly mobile and fly efficiently in low-wind conditions (less than 3 km/h). They avoid resting in windy areas and typically do not fly when wind speeds exceed 2–3 m/s (7–10 km/h) (27, 28). Depending on the species, females may travel from a few hundred meters to several kilometers in search of a blood meal, using a combination of long- and short-range olfaction and visual cues, which include volatile compounds such as carbon dioxide, lactic acid, fatty acid chains, and octenol, as well as colors, shapes, and heat (29).

**Figure 1:**
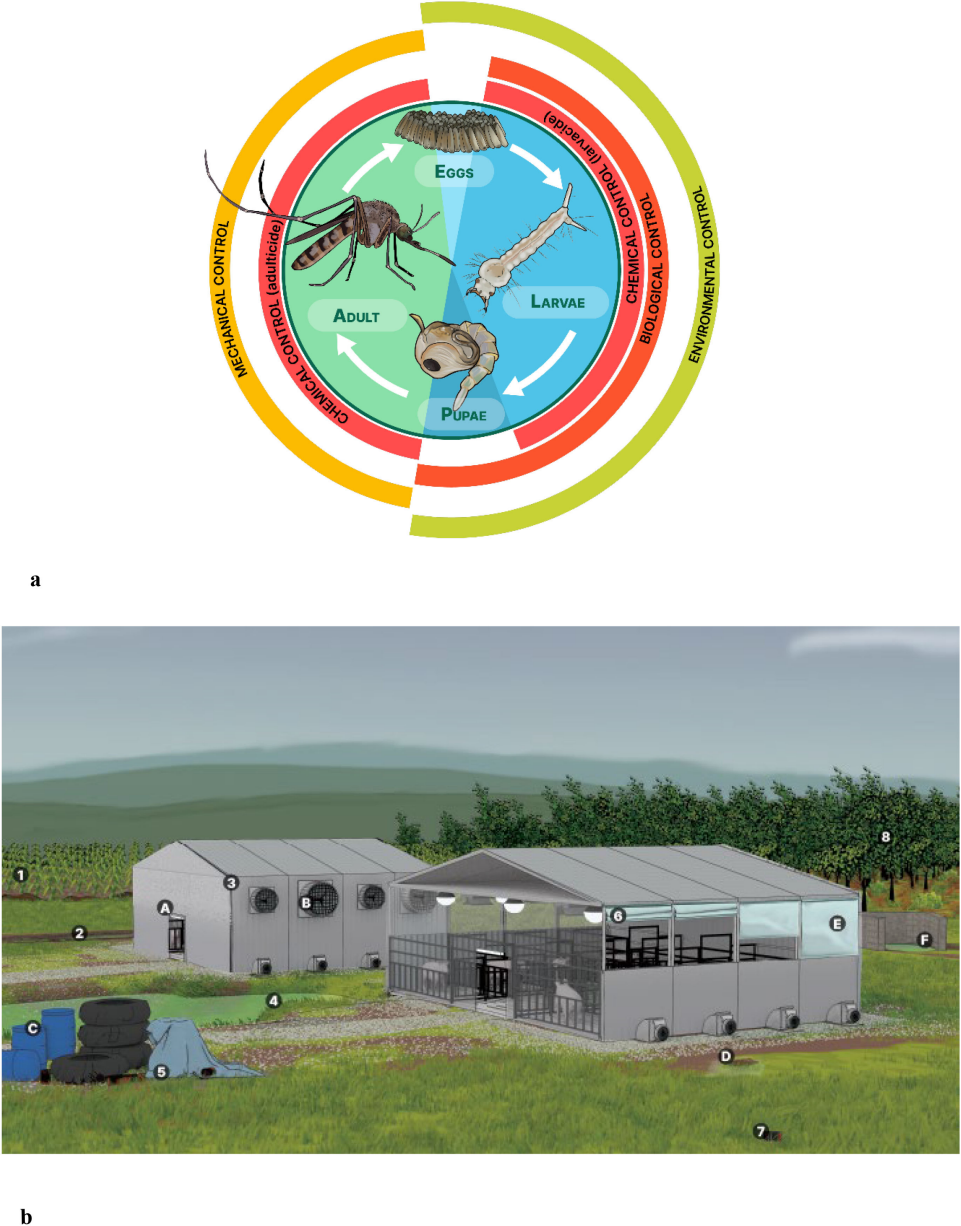
Developmental stage–specific mosquito control strategies in relation to swine facility structure and environment. **(a)** Mosquito life cycle and targeted control strategies by developmental stage. Environmental controls alter habitat conditions making them less suitable for mosquito development, primarily targeting aquatic stages (eggs, larvae and pupae). Biological controls use natural predators to suppress mosquito populations, primarily at the larval and pupal stages. Chemical controls can target larval (larvicides) or adult (adulticides) stages. Mechanical controls involve physical barriers or devices designed to prevent adult mosquitoes from reaching hosts. **(b)** Anatomy of a swine facility. Numbers denote potential larval habitats - areas where water may accumulate and persist long enough to support mosquito breeding. Letters denote corresponding mitigation strategies. Potential larval habitats: (1) Irrigated crops surrounding the facility. (2) Tire tracks filled with rainwater. (3) Clogged gutters or corrugated pipes. (4) Settling ponds or sewage lagoons. (5) Accumulation of garbage, equipment, or spare parts, particularly when covered with tarps. (6) Raised or open curtains can trap water. (7) Discarded containers such as bottles or bags around the facility that may collect water. (8) Adjacent forests which may contain tree holes serving as larval habitats and provide shelter for resting adult mosquitoes. Potential mitigation strategies: (A) Turn off exterior lights in the evening to reduce insect attraction. (B) Install screens on air intake fans to prevent mosquito entry. (C) Cover or properly dispose of barrels and trash around the facility. (D) Fill in potholes and tire tracks to eliminate standing water. (E) Regularly inspect corners and crevices for water accumulation, especially near misters, faucets, and evaporative cooling systems. (F) Ensure proper drainage in cement structures such as the carcass disposal area.

While adult mosquitoes rely on flight and sensory adaptations to locate food and hosts, their life cycle begins in water, making aquatic habitats equally critical for mosquito development and population persistence. Some floodwater species lay eggs on dry ground that is flooded, or in tree rot holes that become inundated during heavy rains. However, many species lay eggs in standing water, such as lagoons, puddles, tire tracks, or clogged gutters, where water persists for days to weeks (30). Mosquito larvae depend on water to develop, making water sources a critical factor for survival and population growth (**Figure 1a**). The transition from egg to adult typically takes 7 to 20 days, depending on species and water temperature. Upon emerging from water, adult mosquitoes rest for several hours as their exoskeletons harden before seeking mates or food (26, 30). Due to their small size and large surface area, mosquitoes are highly susceptible to dehydration and they prefer resting in humid, shaded environments with temperatures ranging from 18 to 26°C, depending on the species (26).

Understanding mosquito physiology, behavior, and life cycle dynamics is essential for designing effective control strategies, insights that form the foundation of an Integrated Mosquito Management approach tailored to swine production systems.

## 3 Integrated mosquito management plan components

An Integrated Mosquito Management (IMM) plan comprises four key components: surveillance, management, record keeping, and education (25). Together these components inform when, where, why, and how to optimally manage mosquito populations below thresholds for economic loss or disease transmission on swine farms. Each facility must establish its own tolerance level for economic impact and tailor its IMM plan to its specific geography, climate, infrastructure, and available resources.

Surveillance identifies the timing and location of species-specific mosquito populations that exceed action thresholds, prompting targeted and timely interventions. Management strategies should be both species- and site-specific to maximize effectiveness while minimizing costs, labor demands, and unintended environmental or animal health consequences. Broad, facility-wide treatments should generally be avoided to conserve resources and reduce risks to pigs, employees, and the surrounding environment. Record keeping is essential for assessing the cost-effectiveness of interventions, which may vary depending on the facility size and characteristics. By integrating surveillance data with swine production records, producers can evaluate intervention outcomes and demonstrate tangible benefits. Education enables producers to select intervention strategies, starting with the most practical or least resource-intensive options and escalating as needed. It also promotes awareness of other benefits of IMM, such as reduced populations of filth or biting flies, improved animal health and welfare, and diminished transmission risk of zoonotic pathogens such as WNV and JEV.

An IMM plan integrates multiple management methods to reduce reliance on a single approach and mitigate the risk of insecticide resistance. These methods fall into four categories (25). Environmental controls modify habitat conditions to make them less suitable for mosquitoes, primarily targeting early-life, aquatic stages (eggs, larvae, and pupae). Biological controls leverage natural predators or pathogens to suppress mosquito populations primarily at the larval and pupal stages. Chemical controls include the application of insecticides, which can target either larval stages (e.g., larvicides) or adult mosquitoes (e.g., adulticides). Mechanical controls use physical barriers or devices to prevent terrestrial-stage, adult mosquitoes from reaching hosts (**Figure 1a**).

The U.S. Environmental Protection Agency (EPA) offers valuable resources on mosquito control methods (31). Additionally, the *“Integrated Mosquito Management Principles for Piggeries”*, developed by the National Vector Management Group in Australia in response to the 2022 JEV outbreak, provides comprehensive guidance on mosquito control in pig farming (22). While these guidelines offer valuable insights, any chemical treatment of pigs or their environment should be carefully evaluated and discussed with a qualified professional.

For vector-borne diseases, controlling vectors to stop pathogen transmission is one of the most effective measures. In the event of a disease outbreak in the U.S., vector control efforts would be carried out at the discretion of county or municipal governments, with specific directives issued by USDA APHIS. This agency will coordinate and collaborate with other entities, including the Centers for Disease Control and Prevention (CDC) and local jurisdictions, to implement vector control strategies. The “*NAHEMS guidelines: Wildlife management and vector control for a foreign animal disease response in domestic livestock*” provide further guidance on the responsible authorities and available vector control methods (32).

### 3.1 Mosquito surveillance

Effective mosquito surveillance is critical for optimizing management strategies. Larval surveillance involves collecting the immature mosquito stages from their aquatic habitats, which then may be treated to prevent adult emergence. Adult surveillance is typically conducted using mechanical suction traps or sticky cards (33). Suction traps attract mosquitoes with odors or lights and can be placed near pigs to exploit the mosquitoes’ natural predilection to seek hosts. Sticky cards, positioned on walls or suspended from ceilings, take advantage of mosquitoes’ habit of resting on vertical surfaces after flying or feeding (30).

Year-round surveillance helps track seasonal fluctuations in mosquito populations and identify areas requiring interventions. Identifying mosquito species present allows entomologists to determine the most effective treatment. For example, floodwater mosquitoes such as *Anopheles* species cannot be controlled at the larval stage, whereas a high abundance of container-breeding species like *Aedes albopictus* or *Aedes aegypti* indicates that small pools of water are accumulating after rainfall and should be eliminated (34). Conversely, an increase in *Culex* mosquitoes suggests a larger, more permanent, nutrient-rich water source that requires targeted treatment (34).

### 3.2 Environmental control methods

Environmental control methods make habitats less suitable for ovipositing mosquitoes. Along with chemical control, environmental methods are among the most commonly recommended insect mitigation strategies. Key environmental controls include optimizing water drainage to eliminate standing water, managing vegetation to reduce breeding habitats, and ensuring proper waste and carcass disposal both inside and outside swine housing facilities (15, 16, 19, 20, 22). Specifically, locating and draining any stagnant pools of water is a recommended control strategy as some mosquito species can lay eggs in as little as one inch of water (20). The Japanese encephalitis Vector Management Group advises sealing or covering water storage containers with 1-mm mesh screening to prevent mosquito oviposition. They also recommend eliminating areas where water remains undisturbed for seven or more days to control larvae (22). Although many environmental control methods are considered “best practices”, there is limited information on specific implementation protocols or their effectiveness. **Figure 1b** illustrates potential larval habitats and corresponding environmental control methods. While these measures can be cost-effective in the long term, they often require significant initial investment, ongoing labor, and coordination among facility managers to maintain effectiveness.

### 3.3 Biological control methods

Biological control methods involve utilizing natural predators of mosquito larvae (e.g., fish, insects, fungus, bacteria) or adult mosquitoes (e.g., bats or birds) to reduce populations. Among these, control efforts targeting the aquatic life states of mosquitoes are typically more effective, as larvae are confined and more accessible compared to highly mobile adult mosquitoes. While the introduction of natural predator species may be feasible around swine premises, these approaches are often impractical (35). Additionally, the effectiveness of these strategies is highly situation-specific, and they should only be implemented after consulting with an entomologist or pest control expert.

Another biological method, the sterile insect technique (SIT), involves releasing sterilized mosquito males to mate with wild females, resulting in infertile eggs and a reduced population over time. Originally developed for New World screwworm (36), SIT has since been applied to *Aedes aegypti* in various locations around the world (37, 38). However, SIT, as well as Wolbachia-based suppression (39) and gene-drive (40) management techniques are species-specific. This represents a challenge in swine production systems, where multiple mosquito species typically co-exist, and eliminating one species will not fully solve the problem. Furthermore, the release of genetically modified organisms is restricted under federal regulations and often has poor public acceptance (40). Biological methods are mostly environmentally friendly and sustainable, but their effectiveness can vary depending on environmental conditions. While these methods hold promise, their application must be tailored, regulated, and carefully integrated within a broader mosquito management framework.

### 3.4 Chemical control methods

Chemical control methods, including larvicides and adulticides can be applied to either the swine housing facility (e.g., baits, fogs, ultra-low volume sprays, premises washes, residual treatments on walls) or directly to the pigs (e.g., injectable or topical). While many commercially available products come with clear instructions and evidence of effectiveness, chemical control is generally the least preferred option. Following label directions in addition to state and local regulations is essential when utilizing chemical control methods (16, 19, 20, 22). These methods are relatively easy to apply and provide rapid results, making them a preferred option for immediate mosquito suppression. However, they incur recurring costs, may require specialized equipment or personnel for proper application, and may have adverse animal and environmental impacts, including insecticide resistance if overused or improperly managed (15, 20, 22).

One valuable resource in the U.S. is the *VetPestX* database (https://www.veterinaryentomology.org/vetpestx), which provides information on state-registered pest management products and offers educational materials for producers, veterinarians, and the public. On a global scale, the World Health Organization (WHO) offers guidance on pesticides and their use to control vectors and pests of public health significance (41). Producers should consult with a veterinarian to ensure the proper use of treatments and compliance with regional regulatory guidelines when applying products directly to animals as some products have lengthy withdrawal times. A well-structured mosquito surveillance strategy can help prevent the unnecessary or excessive use of insecticides, conserving time, money, and resources while slowing the development of insecticide resistance (42, 43).

### 3.5 Mechanical control methods

Mechanical control methods create physical barriers that prevent host-seeking adult mosquitoes from contacting hosts, or modify light sources to reduce attraction (**Figure 1b**). Some methods, such as mosquito nets, have been evaluated and proven effective (44, 45), while others, including double-door entry and exit systems and ventilation designs, require further research to assess their effectiveness in mosquito exclusion. Management strategies like alterations to lighting patterns, such as turning off lights near and inside barns at night or utilizing electric insect removal devices, have been recommended but remain untested for effectiveness (20). These methods provide immediate and highly effective means of protection, but they can be expensive to install and maintain. Additionally, their effectiveness depends on proper design, regular upkeep, and ensuring that gaps or breaches do not occur.

#### 3.5.1 Barriers

While housing pigs in enclosed facilities may help to prevent contact with some types of insects, mosquitoes will seek to enter facilities by targeting openings such as doors, windows, or ventilation systems (**Figure 1b**). Double-door entry and exit systems have been recommended as biosecurity measures for microbial pathogens, but their effectiveness has not been evaluated for insect exclusion (15, 19).

The utilization of insect screens as a mechanical control method has been both recommended and evaluated for effectiveness (15, 22, 44, 45). Schurrer et al., found that commercially available fiberglass mosquito netting with 1-mm square holes affixed to the outside of a naturally ventilated U.S. commercial finishing facility resulted in fewer mosquitoes per room and fewer bites per pig compared to rooms without screens (45). They also demonstrated that the screens had no adverse effects on carbon dioxide levels, relative humidity, or room temperature when properly maintained. Additionally, Dutta et al., conducted a study in a Japanese encephalitis endemic area of India which demonstrated that keeping pigs under insecticide-treated mosquito nets at night reduced the risk of Japanese encephalitis seroconversion in those pigs (44).

#### 3.5.2 Ventilation

Ventilation systems for swine housing facilities have many design elements that can either improve a facility’s biosecurity against insects or present biosecurity vulnerabilities (**Table 1**). Swine housed in facilities with direct access to outdoor air are the least protected from mosquito entrance into their environment. Open-sided barn designs (e.g., curtain sided, air inlet side of tunnel ventilated barns) often provide a more direct path for mosquitoes entering pig spaces compared to fully enclosed barn designs which present mosquitoes with a more circuitous route to pig spaces as they often must navigate passage through a facility’s attic. Furthermore, some enclosed barn designs can accommodate air filtration systems, often designed as a biosecurity measure against microbial pathogens, which would present additional barriers to mosquito entrance into pig spaces (15, 19). However, mosquitoes can access facilities through very small openings (e.g., cracks around doors and windows, between the shutters of idle fans), making even enclosed, filtered barns challenging to completely seal against mosquito entry.

**Table 1:** Ventilation design elements of swine housing facilities and their impact on mosquito populations.

Facility design	Aims of mosquito control methods		
	Minimize mosquito entrance into facility	Reduce mosquito contact with swine	Impair mosquito ability to establish a population
Fully enclosed building with mechanical ventilation and air flow through an attic space			
	In enclosed buildings, *mechanical control methods* offer the greatest opportunity for minimizing mosquito entrance into the facility; however, because mosquitoes can enter facilities through very small openings, building entrance and exit barriers (i.e., doors) and regular air flow patterns are unlikely to be sufficient for complete exclusion of mosquitoes (15, 19, 20, 22, 35, 36).	*Mechanical control methods* (lights and fans) may be strategically utilized to attract mosquitoes away from pigs or deter mosquito contact with pigs (20).	Utilizing *environmental control methods* to decrease mosquito populations on site will augment *mechanical control methods* in limiting mosquito access to pigs (15, 16, 19, 20, 22). Utilization of *biological* and *chemical control methods* to reduce mosquito populations external to the facility could be considered, but may not be necessary if environmental controls are sufficient (15, 16, 19, 20, 22, 41).
Semi-enclosed building with mechanical ventilation and air flow directly into pig space			
	This air flow design may enhance mosquito entrance by passively transporting mosquitoes into the facility with fresh external air.	*Mechanical control methods* (lights and fans) may be strategically utilized to attract mosquitoes away from pigs (20).	Utilizing *environmental control methods* to decrease mosquito abundance on site is critical since these facility types have fewer structural barriers to exclude mosquitos from pig spaces compared to enclosed buildings (15, 16, 19, 20, 22). Utilization of *biological* and *chemical control methods* to reduce mosquito populations external to the facility may be a higher priority on premises with fewer options for *mechanical control methods*, but caution is still urged regarding these control methods due to their environmental impacts (15, 16, 19, 20, 22, 41).
Open building with natural ventilation (no outdoor pig access)			
	While this facility type has fewer inherent barriers to mosquito entrance, *mechanical control methods* such as strategic lighting patterns may be useful for drawing mosquitoes away from the facilities (20).	*Mechanical control methods* (lights and fans) may be strategically utilized to attract mosquitoes away from pigs or disrupt mosquito-pig contact (20).	
Shelter with outdoor pig access			
	If pigs can be confined during periods of peak mosquito activity, there may be an opportunity to utilize mosquito netting as a *mechanical control method* (35).	Pigs with outdoor access are commonly treated with *parasiticides*. Ensuring that applied parasiticides are effective against mosquitoes can be helpful.	

Once mosquitoes enter a barn’s space, typical air speeds in these areas are insufficient to preclude host-seeking flight and feeding behaviors. To reduce host-seeking mosquito flights, air speed must exceed a velocity of 2.2m/s (with a range of 0.8 to 8m/s depending on the mosquito species). To completely inhibit mosquito flight even higher air speeds are required (27). To efficiently deter mosquitoes, wind must consistently blow directly onto pigs at a speed of roughly 5 miles per hour (440 feet per minute (f/m) or more than 2m/s). While the recommended air speed exiting ceiling inlets of mechanically ventilated swine facilities is 800 to 1,000 f/m (4 to 5m/s), allowing air moving at this velocity to blow directly onto pigs is often detrimental to their comfort (46–49). Therefore, to maintain pig comfort, air speeds at the pig level often fall below those required to effectively disrupt mosquito mobility.

## 4 Discussion

While the U.S. swine industry has made significant progress in implementing enhanced biosecurity plans aimed at preventing the introduction of microbial pathogens, the exclusion of insects, particularly mosquitoes, remains insufficiently addressed. Swine farms invest substantial time, labor and resources in biosecurity, including personal protective equipment, chemicals, and disinfection protocols, to prevent the introduction of pathogens to a facility. Despite these efforts, flies, mosquitoes, and other insects continue to move freely in and out of facilities, coming into direct contact with animals, as well as their feed and water sources.

House flies are common in swine facilities and are well-established mechanical vectors of viruses and bacteria. As few as one to five face flies (50), 100 to 200 horn flies (51), or five stable flies per leg (52) can cause economic losses in cattle production. While these flies are not typically problematic on swine farms, they emphasize how even small numbers of biting insects can result in substantial impacts. The number of mosquitoes needed to cause economic damage remains to be quantified for swine or cattle; however, increasingly mosquitoes are recognized not only as vectors of arboviruses but also as significant contributors to mechanical trauma, stress, secondary infections, production losses, and compromised animal welfare. In addition to these threats, the potential incursion or reemergence of *Cochliomyia hominivorax* (New World screwworm) into previously free regions, such as the U.S., further underscores the need for rigorous insect surveillance and control measures, given the devastating consequences this parasite can have on livestock health and productivity. Recent detections in Mexico indicate that the screwworm is advancing north, intensifying the risk of introduction into the U.S. A Brazilian study of myiasis in pigs found that infestations were concentrated on the forehead and ears, sites prone to skin lesions (53). Similarly, in Uruguay, screwworm-induced myiasis was detected in 27 of 618 harvested feral swine (54). Although these cases were associated with mucosal membranes and with anatomical sites prone to skin lesions or wounds from aggressive dominance behavior, infestations can also occur secondary to mechanical trauma caused by other insects, potentially initiating a cycle of repeated injury, parasitism, and worsening tissue damage. Effective biosecurity strategies must account for these insect threats, and reducing mosquito contact with animals should be considered a first line of defense against insect-related biosecurity breaches.

A key reason for the persistent underestimation of mosquito risks may be that their peak activity occurs during crepuscular and nighttime hours, periods when producers and farm workers are typically not present to observe or report mosquito activity. This limited visibility contributes to a perception that mosquitoes are not a significant concern in swine facilities. However, mosquito surveillance is essential to determine whether mosquitoes are present in problematic numbers and to identify their activity patterns, both seasonally and over the course of the day. Notably, the presence of mosquitoes alone does not equate to immediate risk, as not all species are competent vectors. However, when vector species capable of transmitting pathogens are present, the risk to both animal and human health increases substantially (55). For example, in the U.S., *Culex* species are known vectors of WNV and St. Louis encephalitis virus, while *Aedes* species can transmit Zika, dengue, and Eastern equine encephalitis viruses. Understanding which vector species are present in or near swine facilities allows for targeted interventions to reduce the risk of pathogen transmission during periods of viral emergence or outbreak. To support producers, the CDC maintains a surveillance tool (ArboNET) that provides current data on mosquito-borne disease vectors and cases by region (https://www.cdc.gov/mosquitoes/php/arbonet/index.html).

Another contributing factor to underestimation of mosquito impact is the lack of consistent, recognizable indicators of harm, such as changes in animal health, performance or production metrics, or market outcomes. These changes may be subtle, go unrecognized, or may not be systematically recorded. Although data on mosquito abundance in swine facilities remain limited, preliminary findings from our group indicate high mosquito densities during peak seasons, especially in operations located near standing water, manure lagoons, or vegetation (*unpublished data*). Quantifying mosquito burden is essential, not only to demonstrate the potential impact of mosquito presence, but also to inform the design of physical and procedural barriers to limit mosquito intrusion and support the integration of targeted vector control interventions within farm biosecurity plans.

Despite a variety of available mosquito management tools, standardized protocols, validated interventions, and cost-benefit assessments are notably lacking for animal production facilities. This limits the ability of producers to adopt consistent, effective practices tailored to their specific operational needs. Moreover, existing biosecurity frameworks continue to focus primarily on microbial threats without addressing insect exclusion. Incorporating IMM plans into existing enhanced biosecurity site plans represents a proactive approach to mitigating both seasonal mosquito nuisance and the risk of mosquito-borne disease incursions. However, significant knowledge gaps remain regarding which biosecurity measures are the most effective and feasible to implement. Few peer-reviewed studies address mosquito biosecurity on swine premises and most available guidance originates from university extension, government, or industry resources. To close these gaps, there is a need to develop regionally tailored IMM strategies that account for variations in climate, mosquito species composition, and swine production system type. The importance of region-specific risk assessment is illustrated by recent work evaluating the potential introduction of JEV into the U.S. Using a semi-quantitative risk assessment tool, our group found that among seven U.S. regions, only the south, including states such as Texas, Mississippi and North Carolina, had a non-negligible pathway for virus introduction primarily via infected mosquito eggs or larvae in imported used tires. This region is characterized by large feral swine and ardeid bird populations, as well as the presence of some of the country’s highest concentration of commercial swine production. The overall risk estimate for the south was very high, emphasizing the critical need for targeted surveillance and preparedness in high-risk areas. This example underscores how structured risk assessments can inform regionally appropriate mosquito control and biosecurity priorities (56).

Surveillance is a foundational component of any IMM plan, yet mosquito monitoring is rarely implemented in swine production systems. Tools such as light traps, sentinel traps, gravid traps, or even sticky cards can generate data on mosquito abundance and species composition, informing targeted interventions. However, standardized protocols for trap placement, sampling frequency, and data interpretation tailored to swine production environments are needed to enable scalable implementation and adoption.

While insecticides are not a preferred method for routine mosquito control, targeted chemical interventions may warrant further investigation. Currently, to the authors’ knowledge, no commercially available feed additives for swine are labeled for mosquito control, and the efficacy of existing insect growth regulators against mosquitoes remains largely unexplored. Addressing these gaps could expand the range of viable mosquito management options for producers. Alternative methods, such as the use of adulticides in localized areas or the introduction of biological control agents should also be assessed for feasibility, safety, and cost-effectiveness in swine production settings.

The substantial variation in swine facility design further complicates the development of broadly applicable mosquito biosecurity recommendations. Structural features such as pit ventilation systems, evaporative cooling systems, and fan covers can either limit or facilitate mosquito access. Similarly, management practices such as all-in/all-out scheduling, disinfection, and downtime between production cycles, could influence vector presence, but their roles remain largely unquantified. In addition, poor drainage systems, uncovered water storage containers, and improperly maintained waste lagoons can serve as breeding habitats for mosquitoes, increasing the risk of indoor mosquito intrusion and sustained populations around the facility. Facility size also influences feasibility, for example, treating large sewage lagoons may be impractical for some operations due to limited access or high operational costs. Producers must weigh the feasibility and cost-effectiveness of different control strategies. Long-term interventions, such as habitat modification, physical exclusion (netting), or aerial treatments using drones, can offer sustained benefits but are often resource-intensive. Chemical treatments, in contrast, may be more affordable and easier to implement but offer only short-term relief. Mosquito management decisions must balance cost, labor, and efficacy within the context of each facility’s design, capacity, and risk profile. Additionally, the economic impact of biosecurity measures, such as the effect of insect screens on ventilation efficiency and energy use, remains to be evaluated before they can be widely recommended. Producers are understandably concerned about return on investment, especially when interventions may affect airflow, cooling capacity, or operational costs. Economic modeling of IMM interventions, including sensitivity analysis under different facility and climate scenarios, would support cost-effective decision-making. However, these models would require reassessment if a mosquito-borne swine pathogen, such as JEV, were introduced into the U.S.

Climatic and seasonal variability also play a major role in mosquito pressure on swine operations. Warmer temperatures, increased rainfall, and prolonged humid conditions can drive mosquito population growth, particularly in regions where water accumulation and poor drainage coincide with swine production. These factors could expand mosquito ranges in certain parts of the U.S., potentially increasing mosquito pressure on swine facilities in the coming decades.

The potential impact of mosquitoes on swine behavior and welfare is another underexplored area. Incorporating behavioral assessments in future field studies may help identify specific pig responses to mosquito bites, such as restlessness, skin irritation, changes in feeding or lying behavior, and provide a more comprehensive understanding of how insect pressure contributes to production losses and animal welfare concerns.

The purpose of this article is to raise awareness of the often-overlooked threat posed by insects, particularly mosquitoes, in swine production facilities, with a focus on biosecurity breaches resulting from mosquito intrusion into otherwise secure swine housing. The potential consequences of mosquito exposure for farm workers, including nuisance biting and the risk of zoonotic disease transmission, have received little attention in the context of occupational health. From a One Health perspective, enhancing insect-related biosecurity measures not only protects animal health but also contributes to human health and farm worker safety. This article describes practical strategies that swine producers can implement to manage insect presence in their facilities and highlights the growing risks of inaction, including potential impacts on production efficiency, animal welfare, and public health. However, the need for and effectiveness of specific interventions will vary based on several factors such as facility size, building type, geographic location, and existing infrastructure, making one-size-fits-all recommendations inappropriate. Instead, we provide a suite of potential solutions for producers to evaluate based on their individual risk profiles and operational constraints. Whenever possible, insect control should be integrated into the design phase of new facilities or during modifications of existing ones, as this is the most cost-effective opportunity to implement structural and management changes that reduce mosquito access and persistence, supporting long-term biosecurity goals.

The U.S. swine industry has made great strides in developing and implementing biosecurity plans to safeguard the health of their herds, but expanding these plans to include robust mosquito management strategies is both timely and necessary. Validating and quantifying the effectiveness of these measures will help producers make informed decisions, increasing their confidence that mosquito populations, and the risks they pose, can be meaningfully reduced. Developing mosquito management practices tailored to the unique environments of high-risk areas, such as farrowing and wean-to-finish barns, is essential for protecting vulnerable animal populations from pathogen exposure. Integrating IMM components into producer education programs, biosecurity audits, and certification frameworks (e.g., Pork Quality Assurance Plus^®^) could promote adoption and consistency across the industry (21). Ensuring that insect biosecurity becomes a standard part of training and evaluation will help embed these practices into routine herd health management. Strengthening mosquito and insect biosecurity not only supports the health and productivity of U.S. swine herds but also supports producer livelihoods and contributes to a safe, sustainable pork supply for consumers.

## 5 Conclusion

Mosquitoes represent a persistent and underestimated threat to swine health, biosecurity, and welfare in the United States. While biosecurity frameworks have traditionally focused on microbial exclusion, this perspective underscores the critical need to broaden these efforts to include insect threats, particularly mosquitoes, whose presence may facilitate disease transmission, inflict mechanical damage, and contribute to production losses. Their exclusion from swine facilities must be prioritized as a first line of defense, especially as climate variability and global movement of animals and goods continue to reshape vector distributions and emergence risks.

Addressing mosquito-related vulnerabilities requires a shift in how insect biosecurity is perceived and implemented. This includes investing in routine surveillance, developing standardized protocols for vector monitoring and control, and integrating insect management into existing biosecurity plans, producer education, and certification programs. Regionally tailored strategies, informed by facility design, mosquito ecology, and operational constraints, are essential to ensure feasibility and impact. Importantly, proactive insect management can enhance not only animal health and productivity, but also the safety of farm workers and the resilience of the pork production system.

This article calls for renewed attention to the role of mosquitoes in swine production and presents a suite of actionable strategies to support producers and veterinarians in closing this critical gap. As the swine industry continues to evolve in the face of emerging disease threats and environmental change, expanding the scope of biosecurity to include insect exclusion is necessary for sustaining a healthy, productive, and secure pork supply.

## Data Availability

The original contributions presented in the study are included in the article/**Supplementary Material**. Further inquiries can be directed to the corresponding authors.

## Footnotes

1 The 2021 National Animal Health Monitoring System (NAHMS) swine surveys defined small swine enterprises as sites with fewer than 1,000 pigs (total inventory) and large swine enterprises as sites with 1,000 pigs or greater (total inventory).

2 According to the 2021 NAHMS small swine enterprise survey, 46.4 – 73.0% of small swine farms report that they utilize swine housing facilities that allow pigs access to the outdoors, depending on production phase. These housing types include “open buildings with outside access”, “lot with hut or no building”, and “pasture with hut or no building”.

3 In the 2021 NAHMS large swine enterprise survey “total confinement” is defined as: raising hogs in a totally enclosed area. Animals are totally confined inside a building that often has mechanical ventilation. “Mechanical ventilation” is defined as: air flow in a swine area is created using mechanical rather than natural means (such as breezes). For example, air may be pulled in using fans on the side of the building which then flows out through vents in the ceiling. Depending on production phase 87.6 – 99.0% of large swine farms report that they house their pigs in total confinement with mechanical ventilation.

## References

[1] Clampitt C. Pigs, pork, and Heartland hogs: from wild boar to baconfest. Lanham, Maryland: Rowman & Littlefield (2018).

[2] Meyer S. Hogs and pigs history, implications(2023). Available online at: www.nationalhogfarmer.com/market-news/hogs-and-pigs-history-implications (Accessed October 15, 2024).

[3] USDA ERS. Sector at a Glance(2024). Available online at: www.ers.usda.gov/topics/animal-products/hogs-pork/sector-at-a-glance/:~:text=the%20finishing%20phase.-,Industry%20Structure,technological%20innovation%20(figure%202) (Accessed October 16, 2024).

[4] USDA APHIS VS CEAH NAHMS. Swine 2021, part III: reference of management practices on small-enterprise swine operation in the United States, 2021(2022). Available online at: https://www.aphis.usda.gov/livestock-poultry-disease/nahms/studies/swine-2021-small-enterprise-dashboard (Accessed November 5, 2024).

[5] USDA APHIS VS CEAH NAHMS. Swine 2021, part I: reference of management practices on large-enterprise swine operation in the United States, 2021(2024). Available online at: https://www.aphis.usda.gov/swine-2021-part-i-reference-management-practices-large-enterprise-swine-operations-united-states (Accessed November 5, 2024).

[6] Swine Health Information Center. Mosquitos: An emerging threat to swine health(2024). Available online at: www.nationalhogfarmer.com/livestock-management/mosquitos-an-emerging-threat-to-swine-health (Accessed October 16, 2024).

[7] Wadman M. Rude awakening(2023). Available online at: www.science.org/content/article/how-rains-pigs-and-waterbirds-fueled-shocking-disease-outbreak-Australia (Accessed October 16, 2024)., PMID:

[8] Machtinger ET Gerry AC Murillo AC Talley JL. Filth fly impacts to animal production in the United States and associated research and extension needs. J Integr Pest Manage. (2021) 12:41. doi: 10.1093/jipm/pmab026

[9] Zimmerman JJ ed. Diseases of swine. eleventh edition. Hoboken, New Jersey: John Wiley & Sons, Inc (2019).

[10] Lubisi BA Mutowembwa PB Ndouvhada PN Odendaal L Bastos ADS Penrith M-L. Experimental infection of domestic pigs (*Sus scrofa*) with rift valley fever virus. Viruses. (2023) 15:545. doi: 10.3390/v15020545, PMID: 36851759 PMC9964260

[11] World Organization for Animal Health. Terrestrial animal health code: glossary(2024). Available online at: https://www.woah.org/en/what-we-do/standards/codes-and-manuals/terrestrial-code-online-access/?id=169&L=1&htmfile=glossaire.htm (Accessed October 25, 2024).

[12] Dewulf J Van Immerseel F. Biosecurity in animal production and veterinary medicine. Boston, Massachusetts: CABI (2019).

[13] National Pork Board. Animal disease prevention in swine(2024). Available online at: https://porkcheckoff.org/pork-production-management/animal-disease-prevention/ (Accessed October 24, 2024).

[14] USDA APHIS. Swine disease(2024). Available online at: https://www.aphis.usda.gov/livestock-poultry-disease/swine:~:text=Keep%20pigs%20healthy%3A%20Make%20sure,wildlife%20and%20insects%20under%20control (Accessed October 24, 2024).

[15] Pitkin A Otake S Dee S. Biosecurity protocols for the prevention of spread of porcine reproductive and respiratory syndrome virus(2009). Available online at: https://www.aasv.org/wp-content/uploads/2024/04/2007-dee-biosecurity-manual-english-final.pdf (Accessed October 25, 2024).

[16] Secure pork supply(2024). Available online at: https://www.securepork.org/ (Accessed October 25, 2024).

[17] USDA APHIS. Protect our pigs. Available online at: https://www.aphis.usda.gov/animal-disease/swine/protect-pigs?gad_source=1&gclid=Cj0KCQjw4Oe4BhCcARIsADQ0csn-WLPxztTrngFQp-oEBvRqh3rc22PuKPQgSVJ8wcHSCAT7sHyW6XgaAkD7EALw_wcB (Accessed October 24, 2024).

[18] Amass SF. Biosecurity to prevent mechanical transmission of swine pathogens: Threat or fiction? In: Scruton WC Claas S, editors. 2001 Allen D Leman Swine Conference Proceedings (2001). (St. Paul, Minnesota: College of Veterinary Medicine, College of Agricultural, Food and Environmental Sciences, Extension Service, and Swine Center at the University of Minnesota). p. 43–5. Available online at: https://conservancy.umn.edu/server/api/core/bitstreams/19822003-66c7-4a5f-a7d3-518109c3738f/content.

[19] Levis DG Baker R. Biosecurity of pigs and farm security(2011). Available online at: https://porkgateway.org/wp-content/uploads/2015/07/biosecurity-of-pigs-and-farm-security1.pdf (Accessed October 24, 2024).

[20] Rademacher C Greiner L Ramirez B Cohnstaedt L. Integrated pest management in swine production facilities: management of site insect levels to minimize carcass impact(2024). Available online at: https://store.extension.iastate.edu/product/17147 (Accessed March 14, 2025).

[21] Pork Checkoff. PQA Plus Education Handbook Version 5.0 (2022). Available online at: https://www.porkcdn.com/sites/lms/References+and+Resources/PQAv5+Handbook+English+2.8.22.pdf (Accessed November 7, 2024).

[22] Japanese encephalitis Vector Management Group. Integrated Mosquito Management Principles for Piggeries Version 4 (2022). Available online at: https://www.farmbiosecurity.com.au/livestock/pigs/controlling-mosquitoes-around-piggeries/ (Accessed November 7, 2024).

[23] Australian Government Department of Agriculture, Fisheries and Forestry. Japanese encephalitis virus(2025). Available online at: https://www.agriculture.gov.au/biosecurity-trade/pests-diseases-weeds/animal/Japanese-encephalitis (Accessed March 14, 2025).

[24] Dowsett CK Frentiu F Devine GJ Hu W. Japanese encephalitis transmission in Australia: challenges and future perspectives. Med J Aust. (2025) 222:58–62. doi: 10.5694/mja2.52550, PMID: 39648927

[25] World Health Organization. Handbook for integrated vector management(2012). Available online at: https://iris.who.int/bitstream/handle/10665/44768/9789241502801_eng.pdf (Accessed February 7, 2025).

[26] Eldridge B. Mosquitoes, the *Culicidae* . In: Marquardt W, editor. The Biology of Disease Vectors, 2nd edition. Elsevier Academic Press, USA (2005). p. 95–111.

[27] Service MW. Effects of wind on the behavior and distribution of mosquitoes and blackflies. Int J Biometeorol. (1980) 24:347–53. doi: 10.1007/BF02250577

[28] Service MW. Mosquito (Diptera: *Culicidae*) dispersal - the long and short of it. J Med Entomol. (1997) 34:579–88. doi: 10.1093/jmedent/34.6.579, PMID: 9439109

[29] Takken W. The role of olfaction in host-seeking of mosquitoes: A review. Int J Trop Insect Sci. (1991) 12:287–95. doi: 10.1017/S1742758400020816

[30] Foster WA Walker ED. Mosquitoes (*Culicidae*). In: Mullen G Durden L, editors. Medical and Veterinary Entomology. Academic Press, San Francisco, California (2002). p. 203–62.

[31] USDA EPA. Mosquito control(2025). Available online at: https://www.epa.gov/mosquitocontrol (Accessed February 13, 2025).

[32] USDA APHIS. NAHEMS guidelines: Wildlife management and vector control for a foreign animal disease response in domestic livestock(2014). Available online at: https://www.aphis.usda.gov/sites/default/files/FAD-PReP_NAHEMS_Guidelines.pdf (Accessed February 13, 2025).

[33] Cohnstaedt LW Rochon K Duelh AJ Anderson JF Barrera R Su N-Y. Arthropod surveillance programs: basic components, strategies, and analysis. Ann Entomol Soc Am. (2012) 105:135–49. doi: 10.1603/AN11127, PMID: 26543242 PMC4630213

[34] Yee DA Allgood D Kneitel JM Kuehn KA. Constitutive differences between natural and artificial container mosquito habitats: vector communities, resources, microorganisms, and habitat parameters. J Med Entomol. (2012) 49:482–91. doi: 10.1603/me11227, PMID: 22679854

[35] Vinogradov DD Sinev AY Tiunov AV. Predators as control agents of mosquito larvae in micro-reservoirs (Review). Inland Water Biol. (2022) 15:39–53. doi: 10.1134/S1995082922010138, PMID: 35311016 PMC8917826

[36] Knipling EF. Possibilities of insect control or eradication through the use of sexually sterile males. J Econ Entomol. (1955) 48:902–4. doi: 10.1093/jee/48.4.459

[37] O’Neill SL. The use of wolbachia by the world mosquito program to interrupt transmission of *Aedes aEgypti* transmitted viruses. Adv Exp Med Biol. (2018) 1062:355–60. doi: 10.1007/978-981-10-8727-1_24, PMID: 29845544

[38] Birhanie SK Hans J Thieme Castellon J Macias A Casas R Hoang H. Reduction in *Aedes aEgypti* population after a year-long application of targeted sterile insect releases in the west valley region of southern California. Insects. (2025) 16:81. doi: 10.3390/insects16010081, PMID: 39859662 PMC11765725

[39] Werren JH Baldo L Clark ME. Wolbachia: master manipulators of invertebrate biology. Nat Rev Microbiol. (2008) 6:741. doi: 10.1038/nrmicro1969, PMID: 18794912

[40] Shults P Cohnstaed LW Adelman ZN Brelsfoard C. Next-generation tools to control biting midge populations and reduce pathogen transmission. Parasit Vectors. (2021) 14:31. doi: 10.1186/s13071-020-04524-1, PMID: 33413518 PMC7788963

[41] World Health Organization. Pesticides and their application for the control of vectors and pests of public health importance, 6th ed (2006). Available online at: https://iris.who.int/handle/10665/69223 (Accessed February 13, 2025).

[42] Beier JC Keating J Githure JI Macdonald MB Impoinvil DE Novak RJ. Integrated vector management for malaria control. Malar J. (2008) 7:S4. doi: 10.1186/1475-2875-7-S1-S4, PMID: 19091038 PMC2604879

[43] World Health Organization. Management of insecticide resistance among vectors of public health importance: report of the ninth meeting of the Global Collaboration for Development of Pesticides for Public Health([amp]]lrm;2014). Available online at: https://iris.who.int/handle/10665/145673 (Accessed February 13, 2025).

[44] Dutta P Khan SA Khan AM Borah J Sarmah CK Mahanta J. The effect of insecticide-treated mosquito nets (ITMNs) on Japanese encephalitis virus seroconversion in pigs and humans. Am J Trop Med Hyg. (2011) 84:466–72. doi: 10.4269/ajtmh.2011.10-0270, PMID: 21363988 PMC3042826

[45] Schurrer JA Dee SA Moon RD Deen J Pijoan C. Evaluation of three strategies for insect control on a commercial swine farm. J Swine Health Prod. (2006) 14:76–81. doi: 10.54846/jshap/464

[46] Baker JE. Effective environmental temperature. J Swine Health Prod. (2004) 12:140–3. doi: 10.54846/jshap/391

[47] Samuel R Yang X Zangaro C Darrington J. Basic ventilation system design for pork producers(2020). Available online at: https://extension.sdstate.edu/basic-ventilation-system-design-pork-producers (Accessed February 5, 2025).

[48] TeBockhorst K Arora K Dougherty B Kohl K Shouse S. Swine building ventilation system maintenance and troubleshooting tools(2020). Available online at: https://store.extension.iastate.edu/product/16016.pdf (Accessed February 4, 2025).

[49] van Wagenberg AV de Leeuw MTJ. Measurement of air velocity in animal occupied zones using an ultrasonic anemometer. Appl Eng Agric. (2003) 19:499–507. doi: 10.13031/2013.14922

[50] Trout Fryxell RT Moon RD Boxler DJ Watson DW. Face fly (Diptera: *Muscidae*) - biology, pest status, current management prospects, and research needs. J Integr Pest Manage. (2021) 12:5. doi: 10.1093/jipm/pmaa020

[51] Brewer GJ Boxler DJ Domingues LN Trout Frywell RT Holderman C Loftin KM. Horn fly (Diptera: Muscidae) – Biology, management, and future research directions. J Integ Pest Manag. (2021) 12:42. doi: 10.1093/jipm/pmab019

[52] Campbell JB Berry LL. “Economic threshold for stable flies on confined livestock.” In: 1989 Current status of stable fly (Diptera: Muscidae) research. Petersen JJ Greene GL, editors. Chapter 5. Lanham, MD: Miscellaneous Publications of the Entomol Soc Am (1989) 74:18–22. doi: 10.4182/PZJJ5053.74.18

[53] Siqueira JAA Cruz Ubirajara Filho CR Mota Silva TR Rodrigues Freire Lima TA Costa-Junior LM Camara Alves L. Occurrence and anatomical distribution of myiasis caused by *Cochliomyia hominivorax* (Diptera: *Calliphoridae*) in swine. Vet Parasitol Reg Stud Rep. (2020) 22:100481. doi: 10.1016/j.vprsr.2020.100481, PMID: 33308730

[54] Altuna M Hickner PV Castro G Mirazo S Perez de Leon A Arp AP. New World screwworm (*Cochliomyia hominivorax*) myiasis in feral swine of Uruguay: One Health and transboundary disease implications. Parasit Vectors. (2021) 14:26. doi: 10.1186/s13071-020-04499-z, PMID: 33413607 PMC7789611

[55] Humphreys JM Young KI Cohnstaedt LW Hanley KA Peters DPC. Vector surveillance, host species richness, and demographic factors as west Nile disease risk indicators. Viruses. (2021) 13:934. doi: 10.3390/v13050934, PMID: 34070039 PMC8267946

[56] Dixon AL OIiveira ARS Cohnstaedt LW Mitzel D Mire C Cernicchiaro N. Revisiting the risk of introduction of Japanese encephalitis virus (JEV) into the United States - An updated semi-quantitative risk assessment. One Health. (2024) 17:19. doi: 10.1016/j.onehlt.2024.100879, PMID: 39253386 PMC11381889

