# Supplementary material for: Barrier or breach? Assessing swine housing features for mosquito threats
Source: Front Insect Sci. 2025 Sep 4;5:1606259. doi: 10.3389/finsc.2025.1606259 (PMC12443671; doi:10.3389/finsc.2025.1606259)
Supplement: Supplementary file 1 [file Table1.docx]

Supplementary Material

**Supplementary Table S1.** Insect-vectored disease risks to swine by insect Family. Bolded text indicates that the organism (insect vector or pathogen) is present in the United States and an asterisk (*) indicates that infection is inapparent in swine.

# Insect vectors (biological or mechanical) Disease Risk

**Mosquitoes (*Culicidae*)**

**Genus: *Aedes***

**Species: *aegypti*** (1, 2)*,* ***albopictus*** (2, 3)*,*

*camptorhynchus* (4)*, detritus* (3)*,* ***dorsalis*** (2, 3)*,*

***japonicus*** (2, 3)*, kochi* (2, 3)*,* ***nigromaculis*** (3)*,*

***notoscriptus*** (2, 3)*, polynesiensis* (2, 4)*,* ***vexans*** (2, 3,

4)*, vigilax* (2, 3, 4)

# Eastern equine encephalitis virus (2, 4)

Getah virus (4)

Japanese encephalitis virus (2, 3)

***Mycoplasma suis*** (1, 4)

Ross river virus (2, 4)

**West Nile virus*** (2, 4)

**Genus: *Anopheles***

**Species:** *tessellatus* (2, 3)

**Eastern equine encephalitis virus** (4) Japanese encephalitis virus (2, 3) **West Nile virus*** (4)

**Genus:** *Armigeres*

**Species:** *subalbatus* (2)

Japanese encephalitis virus (2, 3)

**Genus: *Coquillettidia*** (2) **Eastern equine encephalitis virus** (2, 4)

**West Nile virus*** (2, 4)

**Genus: *Culex***

**Species:** *annulirostris* (2, 3, 4)*, bitaeniorhynchus* (2, 3,

4)*, fuscocephala* (2, 3, 4)*, gelidus* (2, 3, 4)*,* ***pipiens*** (2,

3, 4)*, pseudovishnui* (2, 3, 4)*,* ***quinquefasciatus*** (2, 3,

4)*, sitiens* (2, 3)*,* ***tarsalis*** (2, 3)*, tritaeniorhynchus* (2, 3,

4)*, vishnui* (2, 3, 4)

Getah virus (2, 4)

Japanese encephalitis virus (2, 3, 4)

Murray Valley encephalitis virus* (2, 4)

Ross river virus (2, 4)

**West Nile virus*** (2, 4)

**Genus: *Culiseta***

**Species:** *annulata* (3)*,* ***inornata*** (2, 3)*,* ***melanura*** (2, 4)

# Eastern equine encephalitis virus (2, 4)

Japanese encephalitis virus (2, 3)

**West Nile virus*** (2, 4)

**Genus:** *Mansonia*

**Species:** *uniformis* (2)

Japanese encephalitis virus (2, 4)

**Genus:** *Mimomyia* **West Nile virus*** (4)

**Genus: *Ochlerotatus* West Nile virus*** (4)

**Genus:** *Verrallina*

**Species:** *funerea*

Japanese encephalitis virus (3)

**Mosquitoes**

(Unspecified genera) **Porcine Reproductive and Respiratory Syndrome virus** (4)

***Mycoplasma suis*** (5)

# Swine pox virus (4)

**Biting flies (*Diptera*)**

***Ceratopogonidae* (*Culicoides*) *Phlebotominae* (sand flies) *Simulidae* (black flies)** (4, 5)

# Vesicular stomatitis virus (4)

**Biting flies *Mycoplasma suis*** (5)

# Swine pox virus (4)

***Stomoxys spp.* (stable fly)** (1) Classical swine fever (hog cholera) (4)

***Mycoplasma suis*** (1, 4)

**Non-biting flies (*Diptera*)**

# *Muscidae* (filth flies) Porcine Reproductive and Respiratory Syndrome virus (4)

***Musca domestica* (house fly)** (4) Classical swine fever (hog cholera) (4)

***Escherichia coli*** (4)

# Hemolytic streptococci (4)

***Salmonella spp.*** (4)

**Non-flying arthropods**

***Ornithodoros spp.* (ticks)** (4) African Swine Fever (4)

***Haematopinus suis* (pig louse)** (4) ***Mycoplasma suis*** (5)

# Swine pox virus (4)

**Table References**

1. Prullage JB, Williams RE, Gaafar SM. On the transmissibility of *Eperythrozoon suis* by *Stomoxys calcitrans* and *Aedes aegypti*. Veterinary Parasitology. (1993) 50(1–2); 125-135. https://doi.org/10.1016/0304-4017(93)90013-D
2. Walter Reed Biosystematics Unit. Mosquitoes. (2025). <http://wrbu.si.edu/vectorspecies> [Accessed February 10, 2025].
3. Auerswald H, Maquart P-O, Chevalier V, Boyer S. Mosquito Vector Competence for Japanese Encephalitis Virus. Viruses. (2021) 13(6):1154. https://doi.org/10.3390/v13061154
4. Zimmerman JJ. (ed.). Diseases of swine, eleventh edition. Hoboken, New Jersey: John Wiley & Sons, Inc. (2019).
5. Iowa State University of Science and Technology. Swine Disease Manual Index of Diseases. (2025). https://vetmed.iastate.edu/vdpam/FSVD/swine/index-diseases [Accessed February 10,

2025].
